# The Uniform System of Accounts for Hospitals and Institutions

**Published:** 1893-11-25

**Authors:** 


### THE UNIFORM SYSTEM OF ACCOUNTS FOR HOSPITALS AND INSTITUTIONS.

We have received the following letter:—

To have succeeded in formulating an uniform system of accounts,* completely meeting the wants of any one class of institutions, would have been a great work; but to have accomplished the same for institutions of all classes and of all sizes is, indeed, a notable achievement. It has been Mr. Burdett's good fortune to solve what has hitherto been regarded as an insoluble problem, and, as often happens in like cases, the solution, in his skilful hands, appears so easy and simple that many a student of such problems will be amazed that it never occurred to himself. Mr. Burdett has been wise enough not to start a brand new system. He adopts the suggestions of the Committee of Hospital Secretaries, embodying the experience of every system in vogue during the last half-century. The explanations are delightfully lucid, and are sufficient to make clear to the most ordinary mind a subject which has puzzled the best accountants for many years past. To one who, like the writer of this notice, has had to struggle year by year through the accounts of hundreds of institutions, each kept on a different system, some full, others meagre, and all more or less muddled, the proof of the practicability of a complete, clear, and uniform system comes as a welcome surprise. Of its manifold advantages there is no need to speak. Amongst the chief of them will be reckoned the possibility of making accurate comparisons between the results of the management of institutions of a similar class, and the intelligent interest which every member of a committee will be encouraged to take in finances. Equally important is the certainty that waste is reduced to a minimum, and fraud rendered well-nigh impossible. The sources of receipts and branches of expenditure, with their details, can be distinguished at a glance, and the forms, illustrating the method of keeping the necessary books, are full and distinct. Not the least serviceable part of the book are the hints on audit, and on tenders and the supervision of contracts.

Mr. Burdett is in agreement with all who have made a

* The Uniform System of Accounts, Audit and Tenders, for Hospitals and Institutions. By HENRY C. BURDETT. The Scientific Press, 428, Strand.

study of the question in considering an audit, made, as is usual, by a chartered accountant alone, as insufficient. He suggests that a sagacious member of the Board of Management should be associated with the accountant in the audit, as one "who is enabled by his knowledge, on going through every item of the accounts, to see that the published statements are so arranged that nothing is suppressed or hidden which ought to be plainly stated." A small committee of two or three would perhaps be more effective, unless the one happens to be so thoroughly competent as to justify the often-expressed confidence in a "committee of one."

We need hardly remind those responsible for the accounts of institutions that an uniform system, however perfect, is useless unless it is adopted generally. Nothing could be in better taste than the author's appeal to managers on this point. "We are all proud of the voluntary system upon which most of the charitable institutions of the United Kingdom are conducted, but if we are to maintain and still further popularise this noble system of helping others, we must certainly prove that the business arrangements of our charitable institutions will compare favourably with the best systems known to men of business throughout the world. The lunatic asylums, being under a Board of Commissioners, are compelled to have one uniform system of accounts, so that the results may be easily compared. Is it too much to ask from the zealous and able conductors of our voluntary charities that they shall spontaneously determine to institute such a system, although they are free from any such supervision or control? Surely not; and our belief is, that as an uniform system of accounts for hospitals and institutions is now forthcoming, it will be gladly adopted by the majority, at any rate, of the managers."

In order to make such a general adoption more feasible, complete sets of account books carrying out the system, suitable to every class of institution, have been brought out by the "Scientific Press," 428, Strand, at which address they may be inspected. With a set of these books, and a copy of this little work, the system may be started at once without the least difficulty. A new era in the accounts of charitable institutions should begin with the new year, and it is to be hoped than an institution which has not adopted the uniform system in 1894 will be conspicuous as an exception. Mr. Burdett has done his part to meet a crying want. It remains now for the managers of institutions to do theirs, and to insist that their accounts shall in future be completely, clearly, and honestly placed before the public in the form which he has drawn out. It will be hard for anyone, who has once looked through the uniform system, not only to be satisfied with, but even to have patience with any other.—The Author of "London Charities."

